# Determination of quasi-primary odors by endpoint detection

**DOI:** 10.1038/s41598-021-91210-6

**Published:** 2021-06-08

**Authors:** Hanxiao Xu, Koki Kitai, Kosuke Minami, Makito Nakatsu, Genki Yoshikawa, Koji Tsuda, Kota Shiba, Ryo Tamura

**Affiliations:** 1 Graduate School of Frontier Sciences, The University of Tokyo, Chiba, 277-8568 Japan; 2 Research and Services Division of Materials Data and Integrated System (MaDIS), National Institute for Materials Science, Tsukuba, 305-0044 Japan; 3 International Center for Materials Nanoarchitectonics (WPI-MANA), National Institute for Materials Science, Tsukuba, 305-0044 Japan; 4 International Center for Young Scientists (ICYS), National Institute for Materials Science, Tsukuba, 305-0044 Japan; 5 Center for Functional Sensor & Actuator (CFSN), Research Center for Functional Materials, National Institute for Materials Science, Tsukuba, 305-0044 Japan; 6 Materials Science and Engineering, Graduate School of Pure and Applied Science, University of Tsukuba, Tsukuba, 305-8571 Japan; 7 RIKEN Center for Advanced Intelligence Project, Tokyo, 103-0027 Japan; 8 John A. Paulson School of Engineering and Applied Sciences, Harvard University, Cambridge, MA 02138 USA

**Keywords:** Chemical engineering, Techniques and instrumentation, Applied physics

## Abstract

It is known that there are no primary odors that can represent any other odors with their combination. Here, we propose an alternative approach: “quasi” primary odors. This approach comprises the following condition and method: (1) within a collected dataset and (2) by the machine learning-based endpoint detection. The quasi-primary odors are selected from the odors included in a collected odor dataset according to the endpoint score. While it is limited within the given dataset, the combination of such quasi-primary odors with certain ratios can reproduce any other odor in the dataset. To visually demonstrate this approach, the three quasi-primary odors having top three high endpoint scores are assigned to the vertices of a chromaticity triangle with red, green, and blue. Then, the other odors in the dataset are projected onto the chromaticity triangle to have their unique colors. The number of quasi-primary odors is not limited to three but can be set to an arbitrary number. With this approach, one can first find “extreme” odors (i.e., quasi-primary odors) in a given odor dataset, and then, reproduce any other odor in the dataset or even synthesize a new arbitrary odor by combining such quasi-primary odors with certain ratios.

## Introduction

For colors, they are always based on only three primary colors—red, green, and blue (RGB)—so that every single color is reproduced by simply mixing the RGB with different ratio. Primary taste sensations of human beings have also been investigated extensively, and are known as sweet, sour, salty, bitter, and umami, although additional ones have been proposed according to the finding of new receptors^1^. In contrast to sight or taste composed of a rather limited kinds of receptors, there are about 400 different kinds of receptors known for the sense of smell with complex cross selectivity between them for human beings. Therefore, scientists have been still exploring and trying to alternatively define primary odors^2,3^. One of the representative examples reported so far is the Henning's odor prism^4^. In the prism, flowery, fruity, putrid, spicy, resinous, and burnt are regarded as six primary odors, and they are assigned at vertices of a triangular prism. However, there is no guarantee that every single odor can be composed by mixing the relevant primary odors, because it is impossible to thoroughly examine how such an approach works for all different kinds of odors. Therefore, defining primary odors where their combination can represent other odors has been a grand challenge for decades.

**Figure 1:**
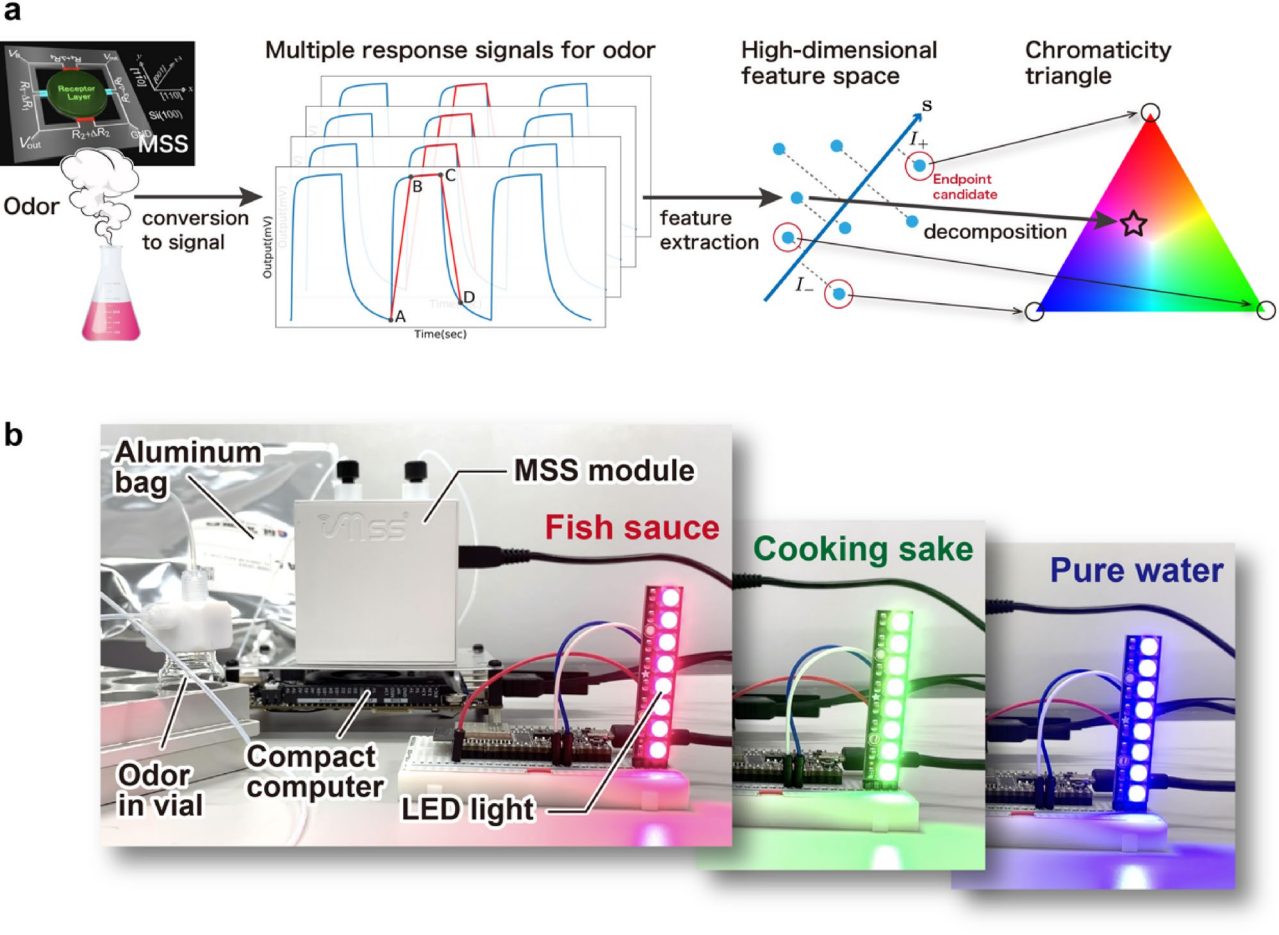
(**a**) Overview of the present quasi-primary odor determination and color representation by combining machine learning and nanomechanical sensing. In the first step, the odor sample is converted to response signals with the MSS. Next, characteristic features are extracted from the signals. By performing machine learning based EP detection, three quasi-primary odors are determined. They are placed at the vertices on a chromaticity triangle. Finally, the other odors are expressed as a mixture of the three quasi-primary odors, resulting in the color representation of each sample. (**b**) Photos of the compact portable device to output a color of odors. The displayed LED color in each photo (from the left to the right) was obtained by measuring fish sauce, cooking sake, and pure water, respectively.

In this paper, we propose an alternative approach to this issue by determining “quasi” primary odors (Fig. 1a). Although it is limited within a given dataset, any odors in the dataset can be represented by the determined quasi-primary odors. For this purpose, characteristic features of various odors are collected with a sensor platform having multiple channels. To convert the sample odors to an analyzable format, a multichannel Membrane-type Surface stress Sensor (MSS) is used^5^. Several advantages of the MSS over a similar type of sensors are its high sensitivity^6^, compact system, and stable operation^7,8^. It was also reported that quantitative analysis and discrimination of odors were achieved with the MSS^9–11^. In the present study, the MSS consists of 12 sensing channels with different response properties was used so that each channel derives different information from the odors. From measured MSS responses, the high-dimensional features for each odor are extracted based on the physicochemical knowledge. The dataset is then analyzed by one of the machine learning techniques, the endpoint (EP) detection method which examines what data points are located at the edge in the high-dimensional feature space to determine quasi-primary odors for the system.

As an application of the present approach to determining quasi-primary odors, the color representation of odors^12^ is demonstrated. Now that quasi-primary odors are available, all the other odors in the dataset are represented as a combination of the quasi-primary odors through quadratic programming from a viewpoint of MSS responses. It indicates that assigning arbitrary colors such as the RGB to three quasi-primary odors enables the color representation of the other odors (Fig. 1a). In recent years, the development of an artificial olfactory system using a multichannel sensor has been reported^5,13–17^. By using such a system, color-based odor visualization was demonstrated in a different manner as we propose here^18–27^. In these works, an odor was represented as a set of multiple colors being displayed at each channel. For example, various dyes and pigments were employed in the case of optoelectronic nose^18,20–23^. An odor sample was converted to a set of colors through the detection of the color changes in each channel. This type of approaches is useful to visually discriminate odors, while it is difficult to reasonably interpret possible reasons for the discrimination as sample odors are independent each other. In contrast, the present approach can connect sample odors via quasi-primary odors of them. Consequently, a color is systematically assigned to an odor, making it possible to find a correlation between sample odors. There are a lot of potential applications for such color representation in the field of food, cosmetics, books, movies, and so on. Since no technique has been established to obtain numeric information of odor itself, memorization, learning, sending, and understanding of odors could even be realized. As an example to easily realize these applications, a compact portable device is developed to output a color of odors in real time, where an MSS module, compact computer, and LED light are connected (see Fig. 1b).

## Results

### Primary odor determination in seasoning odors

**Figure 2:**
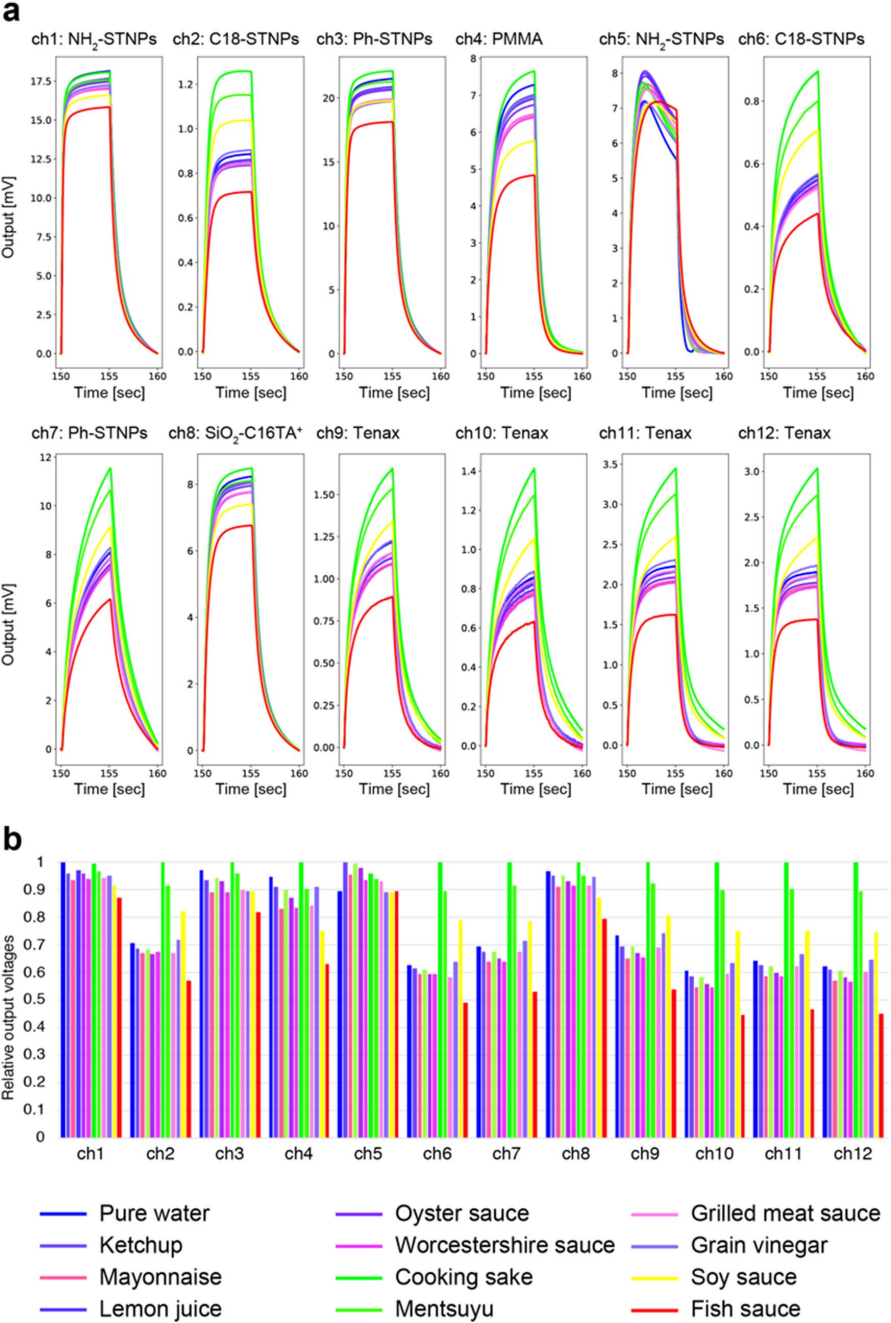
(**a**) Response signals obtained from 12 channels by measuring pure water and 11 types of seasonings. All the response signals are measured repeatedly at an interval of 10 sec (5 sec of odor introduction and 5 sec of purging). The response signals shown here are those recorded between 150 and 160 sec where all the signals are sufficiently stable without any disturbance. The signals are plotted so that the all signal values at 150 sec become zero. (**b**) Relative output voltages for all the 12 samples including pure water and 11 seasonings depending on the channels. For each channel, the output voltages are normalized by the largest one. The output voltages here are simply defined as response signal height.

To demonstrate the present quasi-primary odor determination, we utilized various seasonings. More specifically, 12 types of liquid samples including pure water and 11 seasonings were prepared; the 11 seasonings are ketchup, mayonnaise, lemon juice, oyster sauce, Worcestershire source, cooking sake, mentsuyu, grilled meat sauce, grain vinegar, soy sauce, and fish sauce (detailed ingredients of each seasoning is summarized in Table S1). Figure 2a shows the response signals obtained by measuring the 12 liquid samples with the MSS functionalized with 12 types of receptor materials (see details in Methods section). Although both peak height and signal shape largely vary among all the 12 channels, peak height mainly varies depending on the samples when focus on each channel (signal shape is almost consistent in most cases), indicating that peak height mainly contains important information to differentiate the samples.

From the response signals shown in Fig. 2a, we extracted 48 dimensional features and performed the machine learning-based EP detection to select the three quasi-primary odors for the odor colorization. Here, we set $K=$ 10,000 in the EP detection method (see details in Methods section), and thus the total EP score is 20,000. Table 1 summarizes the ranking of EP scores. The top three samples are fish sauce, cooking sake, and pure water. Since more than half of the total EP scores was voted to the top two odors—fish sauce and cooking sake, they definitely work as the quasi-primary odors in the present case. Then, the EP detection selected pure water as the third quasi-primary odor. A few samples such as grilled meat sauce, oyster sauce, and Worcestershire sauce show much smaller EP scores than the others, indicating that the data points for these odors are located deep inside the 48 dimensional feature space. By performing the EP detection in this manner, the relative positions of each odor in a collected dataset can be understood, specifying the quasi-primary odors for the given samples.

**Table 1:** EP scores, concentrations of primary odors $({w}_{1}, {w}_{2}, {w}_{3})$, and difference $\Delta$ for each odor sample.

Sample	EP score	$({{\varvec{w}}}_{1},{{\varvec{w}}}_{2},{{\varvec{w}}}_{3})$	$\Delta$
Fish sauce	6275	(1.000, 0.000, 0.000)	0.000
Cooking sake	5825	(0.000, 1.000, 0.000)	0.000
Pure water	1917	(0.000, 0.000, 1.000)	0.000
Mentsuyu	1494	(0.181, 0.819, 0.000)	12.654
Soy sauce	1196	(0.501, 0.499, 0.000)	16.692
Grain vinegar	1049	(0.273, 0.210, 0.517)	17.248
Lemon juice	854	(0.219, 0.121, 0.660)	18.699
Ketchup	588	(0.254, 0.160, 0.586)	20.868
Mayonnase	568	(0.509, 0.176, 0.315)	17.270
Grilled meat sauce	132	(0.414, 0.205, 0.380)	10.388
Oyster sauce	80	(0.303, 0.088, 0.609)	14.293
Worcestershire sauce	22	(0.438, 0.097, 0.465)	6.384

Note that the EP score of pure water (the third quasi-primary odor) is relatively lower than those of the top two, and thus, pure water might be replaced by other samples owing to possible external disturbances such as the fluctuations of temperature and humidity during the measurement and/or impurities in the samples. Accordingly, it is important to examine how robust the selection of quasi-primary odors is against the signal noise by our method. For this purpose, Gaussian noise with mean of $0$ and standard deviation of $s$ is adopted artificially to the measured signals, and the effect of the noise is examined systematically. By performing EP detection with several different values of $s$, we evaluated the ranking of EP scores depending on $s$. The results are summarized in Table 2.

**Table 2:**
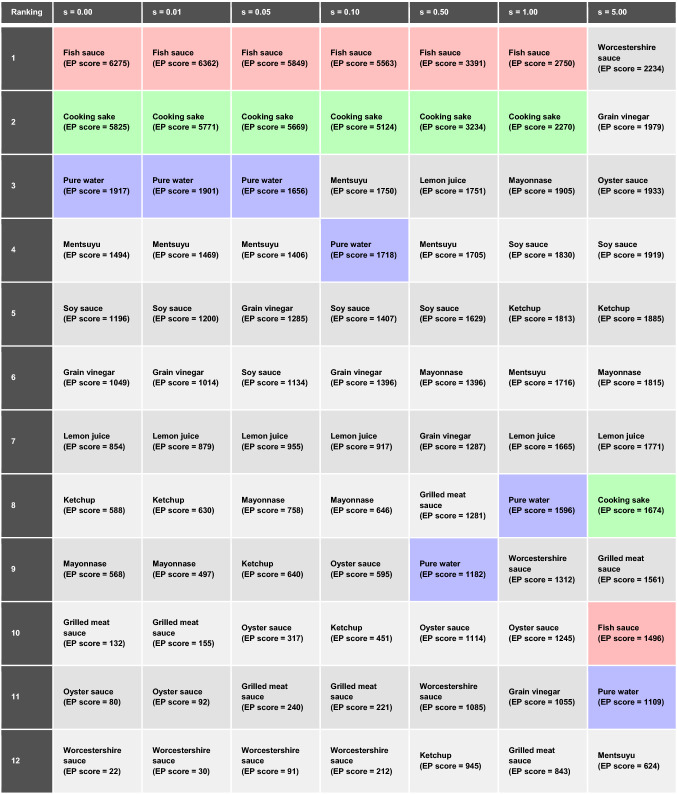
Ranking of EP scores depending on the standard deviation of $s$ for Gaussian noise.

Up to $s=0.05$, the top three samples are the same; the selected quasi-primary odors are fish sauce, cooking sake, and pure water. As $s$ is increased, the EP scores of pure water (3rd) and mentsuyu (4th) become closer. When $s = 0.1$, mentsuyu gets higher value than pure water and is selected as the third quasi-primary odor when three samples are selected. In other words, pure water is much less robust than the other top two samples. In fact the first and second ones remain the same until $s = 1$, indicating that fish sauce and cooking sake are quite robust as quasi-primary odors against signal noise compared to pure water and other samples. The noised signals with $s=0.05, 0.5,$ and $5.0$ are shown in Figs. S1–3. This fact is consistent with the EP scores obtained without these artificial noises. Thus, the EP score would reflect the robustness of the selection of quasi-primary odors against noise.

### Decomposition of odors and its color representation

To decompose odor samples and represent as a mixture of the three quasi-primary odors, the following coefficients $({w}_{1}, {w}_{2}, {w}_{3})$ were calculated by quadratic programming. These coefficients correspond to the composition of the present three quasi-primary odors. In Table 1, these coefficients as well as $\Delta$ values (definition is shown in Eq. (6)), which is the difference between an original signal and composed signal based on $({w}_{1}, {w}_{2}, {w}_{3})$ from a viewpoint of the extracted features, are also denoted for each odor sample. The value of $\Delta$ directly reflects the accuracy of mixing the three quasi-primary odors for the target odor sample. Thus, Worcestershire sauce and grilled meat sauce are more accurately represented by the combination of the three quasi-primary odors.

**Figure 3:**
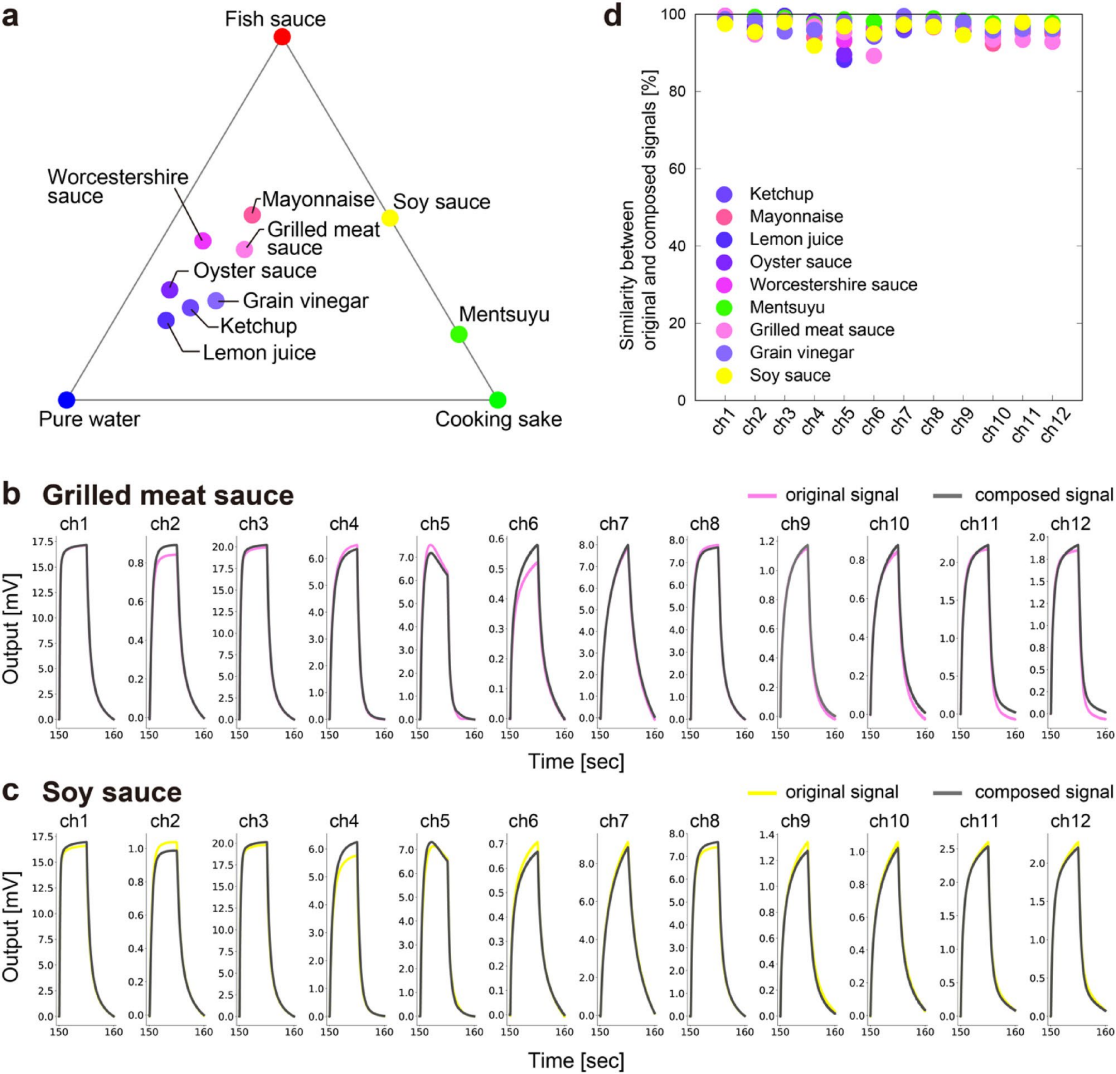
(**a**) Color map of pure water and 11 seasonings. Fish sauce, cooking sake, and pure water are selected as quasi-primary odors. Red, green, and blue are assigned to them, respectively. Original response signals measured with the MSS and the composed signals for (**b**) grilled meat sauce and (**c**) soy sauce. The composed signals are reproduced based on fish sauce, cooking sake, and water using the coefficients $({w}_{1}, {w}_{2}, {w}_{3})$. (**d**) Similarity between original and composed signals for all seasonings except primary odors.

Importantly, the color and the position of each odor sample in the chromaticity triangle were estimated with the three coefficients (Fig. 3a). In this case, the colors of fish sauce, cooking sake, and pure water were assigned to red, green, and blue, respectively. Interestingly, soy sauce and mentsuyu are represented as a binary mixture of fish sauce and cooking sake, while they apparently contain some amount of water. We will discuss possible correlation among receptor material properties, seasoning composition, and the color mapping result afterwards. As the colors assigned to the three quasi-primary odors were arbitrarily selected, the color representation shown here has no specific connection with one of the aspects of the present odor samples. However, the selection of colors which are somewhat correlated with the quasi-primary odors could make this color representation useful to investigate the meaning of resultant colors in the mixture, for example.

As all the odor samples were projected onto the color map, we then confirm how accurately reproduce the original MSS signals with the ones composed on the basis of the present three quasi-primary odors. As shown in Fig. 3b,c as an example, the signals for grilled meat sauce and soy sauce are well-reproduced using corresponding coefficients $({w}_{1}, {w}_{2}, {w}_{3})$ (see Fig. S4 for other samples). In Fig. 3d, the similarity between two signals—original one and composed one—is shown for all the seasonings except the three quasi-primary odors. The similarity is defined by one minus the sum of absolute difference between two signals which is normalized by the integrated value of original one. We found that all the response curves agreed well in each channel. This fact means that the raw response signals of the target odor is reproduced with the response signals of present quasi-primary odors and the three coefficients corresponding to the concentrations of them. This information would be helpful to compose or decompose any odors and to create a new odor from present quasi-primary odor samples.

### Correlation among MSS receptor materials, seasoning components, and color mapping

To discuss and interpret a possible origin of the color mapping in Fig. 3a, we need to carefully consider what factors of receptor materials and seasoning components would dominantly contribute to determining fish sauce, cooking sake, and pure water as the quasi-primary odors. For this purpose, relative output voltages for all the 12 samples including pure water and 11 seasonings were extracted for each receptor material (Fig. 2b). As described, fish sauce and cooking sake were ranked as the first two primary odors. This could be simply because the output voltages for them were the largest/smallest for most of the receptor materials (11 out of 12 receptor materials in both cases). Taking into account that our EP detection selects the one which displays more remarkable features than others, fish sauce and cooking sake are reasonable to be selected. Among other 10 samples, a possible reason why water was selected as the third one could be explained as follows; the other nine seasonings are all composed of water with a wide variety of ingredients (Table S1). Again, since the EP detection picks something which is far from others, “pure” water could be the one to be selected.

For further discussion, a relationship between main component(s) of the quasi-primary odors and inherent properties of the present 12 receptor materials has to be considered. An easy option to begin with is cooking sake because it is mainly composed of water and ethanol (Table S1). The receptor materials which clearly showed the largest output for cooking sake were channels 2, 6, 7, 9, 10, 11, and 12. According to a previous study, C18-STNPs and Ph-STNPs should result in relatively larger responses to ethanol than water because of its hydrophobic nature^9^. Tenax is also known as a hydrophobic material so that it preferentially captures ethanol more than water^28^. On the other hand, the enhanced response to water in the case of channels 1, 4, 5, and 8 seems to be reasonable as well. NH_2_-STNPs, PMMA, and SiO_2_-C16TA^+^ are rather hydrophilic than C18-STNPs and Ph-STNPs so that they showed much larger water-to-ethanol response ratio.

In contrast to these two quasi-primary odors, we should discuss fish sauce from a different angle. As pure water is already assigned at one of the vertices of the chromaticity triangle in Fig. 3a, other components should be focused on here. Fish sauce is known to contain many kinds of chemical compounds which are formed in the process of fermentation. Taking account of the fact that only NH_2_-STNPs showed relatively large response to fish sauce (Fig. 2b), electrostatic interaction between amino groups on NH_2_-STNPs and chemical compounds which have a structure to function as an acid could affect the present result. Since fish sauce contains various amino acids^29^, these species possibly contributed to generating the large response. Interestingly, the normalized responses for channels 2, 6, 7, 9, 10, 11, and 12, *i.e.*, C18-STNPs, Ph-STNPs, and Tenax, imply an important point; relative output ratio among fish sauce, soy sauce, mentsuyu, and cooking sake is all the same. Furthermore, this tendency is clearly seen in the color map in Fig. 3a. This result where these four samples are all on the same line could be interpreted as follows; same as fish sauce, soy sauce is also produced through a fermentation process which uses alcohol in the manufacturing process, and mentsuyu contains soy sauce as its component, while cooking sake is neither fermented nor mixed with soy sauce. This is also supported by the fact that mayonnaise, Worcestershire sauce, and grilled meat sauce are located closer to fish sauce than others because they all contain some components such as soy sauce which is fermented. In contrast, lemon juice, which is more like water, is located closer to pure water.

### Real-time color conversion device

In the present approach, if the quasi-primary odors were determined in advance, various odors can be instantly converted to display colors by only performing quadratic programming, realizing real-time odor colorization. To prove this idea, we created a compact portable device to display a color of odors using a compact computer, MSS module, and LED light (Fig. 1b). In our implementation, fish sauce, cooking sake, and pure water, which were determined as the quasi-primary odors as described, were assigned as red, green, and blue, respectively.

**Figure 4:**
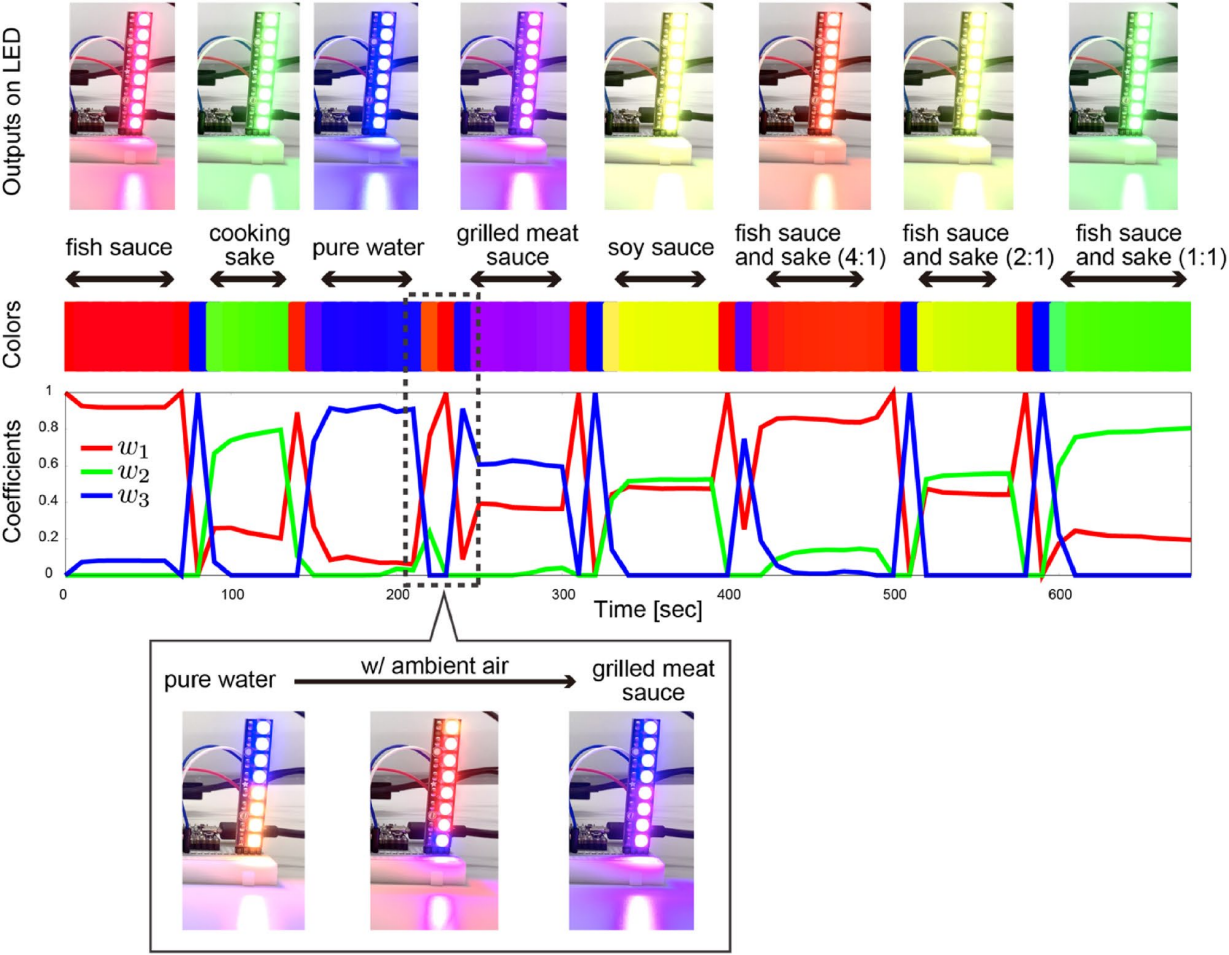
Outputs on LED light, colors, and time dependent change of coefficients $({w}_{1}, {w}_{2}, {w}_{3})$ during the real-time measurement. The samples are measured in the following order: fish sauce, cooking sake, pure water, grilled meat sauce, soy sauce, and three mixtures of fish sauce and cooking sake (concentrations are 4:1, 2:1, and 1:1). A bottom panel shows the change of LED lights when the sample is switched from pure water to grilled meat sauce. The temporal color such as orange/red, which is due to the contamination by ambient air, is observed.

A movie of the real-time measurement is available in the Supporting video 1. In the former half, the real-time color conversion was performed in the following order: fish sauce, cooking sake, pure water, grilled meat sauce, and soy sauce. The outputs on the LED light, colors, and time dependent change of the coefficients $({w}_{1}, {w}_{2}, {w}_{3})$ are shown in Fig. 4. When the samples were switched from one to another, some amount of ambient air was introduced into the measurement system together with sample odors, inducing the large fluctuation of the coefficients (see the bottom panel in Fig. 4). This fluctuation also resulted in the temporal color change to the one which was different from the color of a target odor. After that, the signals reached a stable state to show a reasonable color which is consistent with the one shown in Fig. 3a. Furthermore, in the latter half, three mixtures consisting of fish sauce and cooking sake with different concentrations (4:1, 2:1, and 1:1) were also measured for further validation. We confirmed that these colors are the mixed color of red and green, *i.e.*, the colors of fish sauce and cooking sake, reflecting the result of the color presentation show in Fig. 3a.

## Discussion

In summary, an alternative approach to defining primary odors was proposed, that is, “quasi” primary odors that can represent any other odor in a given dataset by their combination with certain ratios. To visually demonstrate this approach, we utilized an analogy to the well-known RGB model. The three primary colors were assigned to the selected quasi-primary odors, realizing that other odors were expressed with a color depending on the composition of the quasi-primary odors. Furthermore, we developed a compact portable device where our algorithm is implemented to demonstrate a real-time odor colorization.

It should be noted that the number of quasi-primary odors is not limited to three but can be set to an arbitrary number. The color representation is also performed in a straightforward manner when more than three samples are selected as quasi-primary odors. Table S2 shows the colors of each odor sample when four quasi-primary odors using the ranking of EP scores (*i.e.*, fish sauce, cooking sake, pure water, and mentsuyu) are selected and the color of mentsuyu is defined as yellow. If more quasi-primary odors in the collected dataset are determined, high-dimensional analysis of odors can be performed without limitation. Moreover, our approach is not limited to MSS used in this study and can be used universally for other sensor arrays^15,30–33^, for which different quasi primary odors may be selected. In any case, it should be always noted that quasi-primary odors are valid only in the given dataset. To find a “real” primary odor, we need to examine all the combinations of odors and designated sensors, which is technically impossible. It is also important to note that the “primary” odors are defined in relation to receptors; “primary odors” for human being are not necessarily the same for other living things having different receptors and this is also the case for different sensors.

## Methods

### Materials

Octadecyltriethoxysilane (ODTES; Tokyo Chemical Industry Co., Ltd., > 85.0%), 3-aminopropyltriethoxysilane (APTES; Sigma-Aldrich Inc., > 98%), triethoxyphenylsilane (TMPS; Tokyo Chemical Industry Co., Ltd., > 98.0%), titanium tetraisopropoxide (TTIP; Tokyo Chemical Industry Co., Ltd., purity N/A), aqueous ammonia solution (NH_3_ aq.; Kanto Chemical Co., Inc., 28.0–30.0%), octadecylamine (ODA; Aldrich, Inc., 97.0%), and isopropyl alcohol (IPA; Wako Pure Chemical Industries, Ltd., 99.7%) were used for the synthesis of silica/titania hybrid nanoparticles (STNPs). Cetyltrimethylammonium chloride (C16TAC; Tokyo Chemical Industry Co., Ltd., > 95.0%), tetraethoxysilane (TEOS; Tokyo Chemical Industry Co., Ltd., > 97.0%), and methanol (MeOH; Kanto Chemical Co., Inc., 99.8%) were used for the synthesis of silica-cetyltrimethylammonium cation (SiO_2_-C16TA^+^) hybrid. Poly(methyl methacrylate) (PMMA; Sigma-Aldrich Inc.), Tenax TA 20–35 (GL Sciences), and Tenax TA 60–80 (GL Sciences) were used as receptor materials. *N,N*-dimethylformamide (DMF; Kanto Chemical Co., Inc., > 99.5%), 1,1,2,2-tetrachloroethane (TCE; Wako Pure Chemical Industries, Ltd., > 97.0%), and toluene (Kanto Chemical Co., Inc., > 99.5%) were used as solvents to prepare solutions or suspensions of receptor materials for spray coating and inkjet spotting. All chemicals were used as received. MilliQ water was used (Merck MilliPore) as a solvent to prepare a solution of receptor materials and as water vapor.

For sensing measurements, commercially available 11 types of seasonings were purchased: ketchup (Tableland Co., Ltd.), mayonnaise (Kewpie Corp.), lemon juice (Pokka Sapporo Food & Beverage Ltd.), oyster sauce (Tomato Corp.), Worcestershire source (Ikari Sauce Co., Ltd.), cooking sake (Hinode Holdings Co., Ltd.), mentsuyu (Wadakan Corp., Japanese noodle soup base), grilled meat sauce (Ikari Sauce Co., Ltd.), grain vinegar (Tamanoi Vinegar Corp.), soy sauce (Wadakan Corp.), and fish sauce (Allied Corporation Co., Ltd., nam pla).

### Synthesis of nanoparticles

STNPs with various surface functionalities were synthesized by means of a multi-step nucleation controlled growth method which we reported previously with some minor modifications^34^. Briefly, five starting solutions (Solutions A–E) were prepared. Detailed composition of each solution is summarized in Table S3. The Solutions A, B, C, and D were individually flowed in perfluoroalkoxyalkane (PFA: 1.0 mm inner diameter, 1/16 inch outer diameter, product of YMC Co., Ltd.) tubes with a syringe pump (CXN1070, product of ISIS, Co., Ltd.) at 10 mL/min. Two of the starting solutions A, B, and C, D were mixed respectively in a polytetrafluoroethylene (PTFE) fluidic channel with a Y shape junction (the channel cross section of approximately 1 mm^2^, KeyChem mixer, product of YMC Co., Ltd.). After that, resultant two reaction solutions, *i.e.*, Solutions A + B and C + D, were mixed in the second fluidic channel placed just after the first two fluidic channels. The first and second fluidic channels were connected with 10 cm PFA tubes. Then, the mixture composed of all the starting solutions A–D was flowed through a PFA tube with 70 cm in length and was added into the Solution E under magnetic stirring. After the addition, the final reaction solution was aged at room temperature for 24 h. Finally, a slightly turbid suspension was obtained. We used 3-aminopropyl STNPs (NH_2_-STNPs) for channels 1 and 5, octadecyl STNPs (C18-STNPs) for channels 2 and 6, and phenyl STNPs (Ph-STNPs) for channels 3 and 7.

The SiO_2_-C16TA^+^ hybrid was synthesized by means of surfactant-templated nanoparticle synthesis according to the reported procedure^35^, and used for channel 8. Briefly, C16TAC (0.211 g), ultrapure water (17.7 g), methanol (175 mL), and NH_3_ aq. (7.20 g) were mixed in a polypropylene bottle with a volume of 500 mL. The mixture was hand-shaken for 3 sec to obtain a clear solution. To initiate particle formation reaction, TEOS (1.84 mL) was added to the solution quickly, followed by another hand-shaking for 3 sec. Then, the reaction solution was aged at room temperature for 24 h. The solid product was centrifuged, washed with MeOH three times, and carefully transferred into a certain amount of ultrapure water without drying to prepare 1 g/L suspension for inkjet coating.

Commercially available porous polymer beads (Tenax TA) with 20–35 mesh in particle size for channels 9 and 11, and with 60–80 mesh in particle size for channels 10 and 12, and commercially available polymer, PMMA for channel 4 were also used as a receptor material in this present study.

### Characterization

Fourier Transform-infrared (FT-IR) spectra were measured using a Nicolet 4700 FT-IR spectrometer (Thermo Fisher Scientific Inc.) at a resolution of 2.0 cm^−1^ and in the range from 4000 to 400 cm^−1^. The sample powder was homogeneously mixed with KBr (Specac Ltd.), and then the mixture was pressed to form a KBr disk for the transmission measurements. Scanning electron microscope (SEM) images were obtained using a Hitachi Ultrahigh Resolution Scanning Electron Microscope SU8000 at an accelerating voltage of 10 kV. Prior to each measurement, samples were coated with a few nanometers of platinum. These characterizations are the same with previous work, and then these descriptions have already been described in our previous publication^9^.

Thermogravimetric-differential thermal analysis (TG–DTA) curves were recorded on a SII EXSTAR 6000 TG/DTA6300 at a heating rate of 10 °C/min under a constant air flow. $\alpha$-alumina powder was used as a reference material to obtain DTA curves.

### Spray coating of NH_2_-STNPs onto MSS

The detailed fabrication procedure of the MSS chip was described in our previous reports^6^. NH_2_-STNPs suspensions were spray-coated onto the surface of MSS by using a spray coater (rCoater, product of Asahi Sunac Co.). For the preparation of NH_2_-STNPs suspension, as-synthesized NH_2_-STPNs suspension was centrifuged at 9000 rpm for 10 min. The resulting sediment was carefully washed with IPA several times and then IPA/water mixture (vol/vol = 3/5) was added. The concentration of NH_2_-STNPs suspension was set at approx. 1 g/L. Before spray-coating, the suspensions were fully ultrasonicated to get the nanoparticles dispersed as much as possible (some aggregates were still recognized).

Then, the suspension was loaded in a syringe and was flowed through a PTFE tube at 3 mL/min by using a syringe pump (YSP-201, product of YMC Co., Ltd.). The suspension was introduced into a spray nozzle and then was sprayed with the help of two types of carrier air (atomizing air: 0.030 MPa, patterning air: 0.030 MPa) to form homogeneous droplets. The MSS was mounted on a stage which was heated at approx. 100 °C to quickly evaporate the droplets. The stage was moved back and forth, while the spray nozzle was also moved from left to right at 15 mm/sec with 0.3 mm pitch. The distance between the spray nozzle and stage was set at 100 mm. The coating process was repeated to obtain coating thickness of around 1 μm. The spray coating method is the same with previous work, and then these descriptions have already been described in our previous publication^9^.

### Inkjet coating of receptor materials onto MSS

STNPs, SiO_2_-C16TA^+^, PMMA, and a series of Tenax TA were deposited onto the surface of MSS by inkjet spotting. An inkjet spotter (LaboJet-500sp) and a nozzle (IJHBS-300) were purchased from MICROJET Corporation. Each receptor material was dissolved or dispersed in selected solvents as presented in Table S4. For the preparation of STNPs suspensions, as-synthesized STNPs were washed with IPA several times as described above and then dispersed in each medium with the concentration of approx. 1 g/L. The resulting solutions were dropped onto each channel of the MSS by the inkjet spotter to form a receptor coating. The injection speed and volume of a droplet were fixed at approx. 5 m/sec and approx. 300 pL, respectively. The number of shots were summarized in Table S4. A stage of the inkjet spotter was heated at a certain temperature to dry dispersants (Table S4).

### Detailed procedure and conditions for the sensing experiments

In the present study, the MSS chips coated with the receptor materials were placed in a PTFE chamber and the chamber was carefully sealed with O-rings. The chamber was connected to a sample flow system consisting of a pump, a switching valve, a purging gas line (*i.e.*, accelerating desorption of adsorbents), a glass vial for a target sample liquid as illustrated in Fig. S5. The vapors of target samples at the headspace of the vial were introduced by flowing a carrier gas. Pure nitrogen gas was used as carrier and purging gases. In the present case, a given amount of sample liquid (10 mL) was added into 20 mL vial sealed with an O-ring and a custom PTFE vial lid, and two PTFE tubes was connected into the headspace of the vial through the custom lid (Fig. 1b). One end of the PTFE tube was connected to an aluminum bag containing pure nitrogen gas and the other end of the PTFE tube was connected to the switching valve. The flow rate of the pump was set at 30 mL/min, and the switching valve was switched every 10 sec to perform a sample introduction-purging cycle. Data were measured with a bridge voltage of –1.0 V, and recorded with a sampling rate of 100 Hz.

In our measurements, we kept the temperature of the sensors and the samples as constant as possible, because temperature is one of the factors that affect signals. To obtain reproducible signals, all the response signals were measured under the same constant room temperature.

When the quasi-primary odors are selected by endpoint detection, we used the averaged value of five peaks in between 150 and 200 sec. On the other hand, for real-time conversion devise, the last complete peak obtained at that moment was used as it is.

### Features of each response in a signal

As features of a signal in EP detection, the four parameters were extracted from a response in a signal measured with the MSS: The parameter 1 is defined by the slope of points A and B shown as the second panel from left in Fig. 1a, and this is related to the adsorption process of odor. The slope of points B and C is used as the parameter 2, and the information of quasi-equilibrium state would be reflected. The parameter 3 is defined by the slope of points C and D which is relating to the desorption process of odor. Finally, the parameter 4 is the height of the signal, and this includes the information of the adsorption capacity of each receptor material. Here, we fixed the time differences between points A and B as 0.5 sec, which is also used for points C and D. We prepared 12 types of receptor materials, and thus, one odor sample is represented by 48 parameters (*i.e.*, four features times 12 channels). This is the 48 dimensional feature of each odor.

### Determination of quasi-primary odors by EP detection

To determine the quasi-primary odor samples, the EP detection method inspired from Pixel Purity Index (PPI) algorithm^36^ is performed for the 48-dimensional features of odors obtained by MSS measurements. Let $N$ be the number of odor samples, and ${\mathbf{x}}_{i}$ is the vertical vector containing the 48-dimensional features of the $i$th odor sample. The feature matrix $X$ is defined as

1 $$ X = \left( {{\mathbf{x}}_{1} , {\mathbf{x}}_{2} , \ldots , {\mathbf{x}}_{N} } \right), $$

and each row in $X$ is standardized so that the mean and deviation are 0 and 1, respectively. First, we set a large number $K$ and generate a set of $K$ random unit vectors $\left\{ {{\mathbf{s}}_{k} } \right\}_{k = 1, \ldots ,K}$. The dimension of each ${\mathbf{s}}_{k}$ is the same with that of feature vector. A vector ${\mathbf{n}} = \left\{ {n_{i} = 0} \right\}_{i = 1, \ldots ,N}$ is prepared to contain scores for EPs called EP score, which counts how many times each data is regarded as an extremum. We perform the following iteration $K$ times.

1. The all data are projected on ${\mathbf{s}}_{k}$, that is, we calculate ${\mathbf{y}} = X^{{\text{T}}} {\mathbf{s}}_{k}$ which is the coordinates of each odor sample on the direction of ${\mathbf{s}}_{k}$.
2. The indices of the maximum and the minimum values of ${\mathbf{y}}$ are obtained:
  2 $$ I_{ - } = {\text{arg min}}_{i} {\mathbf{y}}, $$
  3 $$ I_{ + } = {\text{arg}} {\text{max}}_{i} {\mathbf{y}}. $$
3. The value of the selected element in ${\mathbf{n}}$ is updated as:
  4 $$ n_{{I_{ - } }} \to n_{{I_{ - } }} + 1, $$
  5 $$ n_{{I_{ + } }} \to n_{{I_{ + } }} + 1, $$
  that is, one point is added to EP score for the selected element $I_{ - }$ and $I_{ + }$.

The example of this iteration is shown as the third panel from left in Fig. 1a. If $K$ is large enough, the odor sample having large EP score ${n}_{i}$ would be located at the end in the high dimensional feature space. Thus, we select some odors with larger EP scores as quasi-primary odors. If we consider the visualization of odors (i.e., colorization in the present study), three odor samples with larger ${n}_{i}$ are determined as quasi-primary odors.

### Composition of an odor by quasi-primary odors

To compose the target odors as a mixture of quasi-primary odors, we search for the linear combination of standardized feature vectors of determined quasi-primary odors so that the feature vector of the target odor is realized as far as possible from a viewpoint of responses. Here, we consider the case that the number of quasi-primary odors is $M$, which are selected by EP detection. The indices of the selected EP samples are put as $\left\{ {{\mathbf{{\text{e}}_{j} }} } \right\}_{j = 1, \ldots ,M}$. The features of quasi-primary odors are $\left\{ {{\mathbf{x}}_{{{\text{e}}_{j} }} } \right\}_{j = 1, \ldots ,M}$, and $\left\{ {w_{j} } \right\}_{j = 1, \ldots ,M}$ are the coefficients of linear combination of the feature vectors of quasi-primary odors. By performing the quadratic programming^37^, we calculate these coefficients for the feature vector of target odor ${\mathbf{x}}$ so that the following difference $\Delta$ becomes minimum value:

6 $${\Delta ={{\left\| {\overset{\lower0.5em\hbox{$\smash{\scriptscriptstyle\rightharpoonup}$}} {{\mathbf{x}} - \mathop \sum \limits_{j = 1}^{M} w_{j} {\mathbf{x}}_{{{\text{e}}_{j} }}} } \right\|^{2} }},}$$

under the conditions of $w_{j} \ge 0$ and $\mathop \sum \nolimits_{j = 1}^{M} w_{j} = 1$. The former condition is necessary to achieve positive mixture of quasi-primary odors, because the negative concentration of quasi-primary odors is impossible. Furthermore, the latter condition is imposed to directly express concentrations of quasi-primary odors for the target odor.

Let the color of each quasi-primary odors be defined by the RGB value as $\left[ {r_{j} , g_{j} , b_{j} } \right]$ for $j = 1, \ldots ,M$. In this case, using $\left\{ {w_{j} } \right\}_{j = 1, \ldots ,M}$, the RGB value of the target odor can be represented by $\left[ {\mathop \sum \nolimits_{j = 1}^{M} w_{j} r_{j} , \mathop \sum \nolimits_{j = 1}^{M} w_{j} g_{j} , \mathop \sum \nolimits_{j = 1}^{M} w_{j} b_{j} } \right]$. If the three quasi-primary odors are selected ($M = 3$), the position on the chromaticity triangle for the target odor is obtained as $\left( {w_{1} /2 + w_{2} , \sqrt 3 w_{1} /2} \right)$, and using this, the color of each odor sample is obtained by the RGB color model.

## Supplementary Information

Supplementary Files.

Supporting video 1.
