# Supplementary material for: Determination of quasi-primary odors by endpoint detection
Source: Sci Rep. 2021 Jun 8;11:12070. doi: 10.1038/s41598-021-91210-6 (PMC8187439; doi:10.1038/s41598-021-91210-6)
Supplement: Supplementary file 1 — Supplementary Files. [file 41598_2021_91210_MOESM1_ESM.docx]

SUPPORTING INFORMATION for

Determination of quasi-primary odors by endpoint detection

Hanxiao Xu^1,2^, Koki Kitai^1,3^, Kosuke Minami^4^, Makito Nakatsu^5^, Genki Yoshikawa^5,6^, Koji Tsuda^1,2,7^, Kota Shiba^*5,8^, and Ryo Tamura^*1,2,3^

^1^Graduate School of Frontier Sciences, The University of Tokyo, Chiba 277-8568, Japan

^2^Research and Services Division of Materials Data and Integrated System (MaDIS), National Institute for Materials Science, Tsukuba 305-0044, Japan

^3^International Center for Materials Nanoarchitectonics (WPI-MANA), National Institute for Materials Science, Tsukuba 305-0044, Japan

^4^International Center for Young Scientists (ICYS), National Institute for Materials Science, Tsukuba 305-0044, Japan

^5^Center for Functional Sensor & Actuator (CFSN), Research Center for Functional Materials, National Institute for Materials Science, Tsukuba 305-0044, Japan

^6^Materials Science and Engineering, Graduate School of Pure and Applied Science, University of Tsukuba, Tsukuba 305-8571, Japan

^7^RIKEN Center for Advanced Intelligence Project, Tokyo 103-0027, Japan

^8^John A. Paulson School of Engineering and Applied Sciences, Harvard University, Massachusetts 02138, United States

**
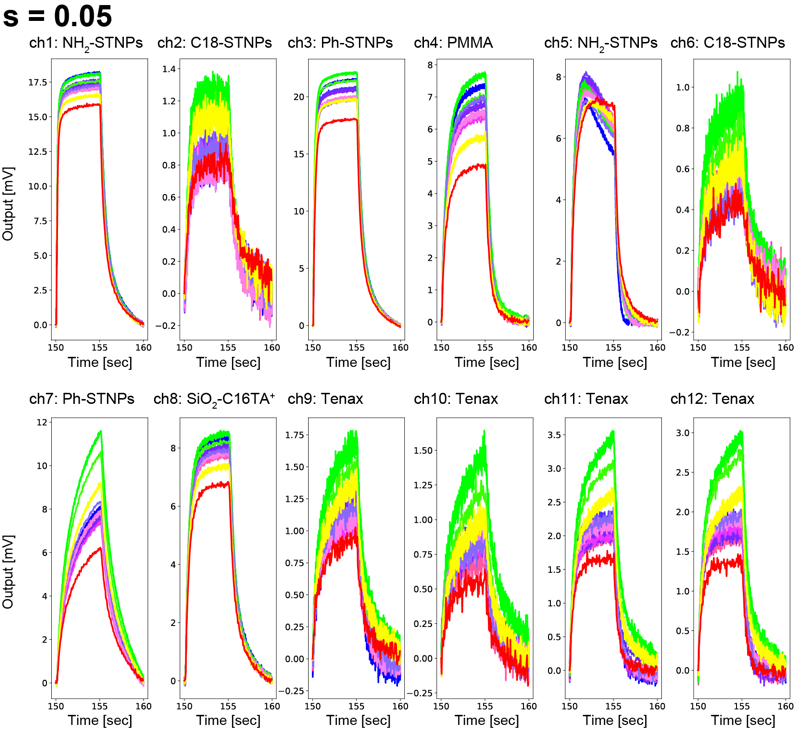
**

**Figure S1**. Response signals adding Gaussian noise with mean of 0 and standard deviation of $s=0.05$ obtained from 12 channels.

**
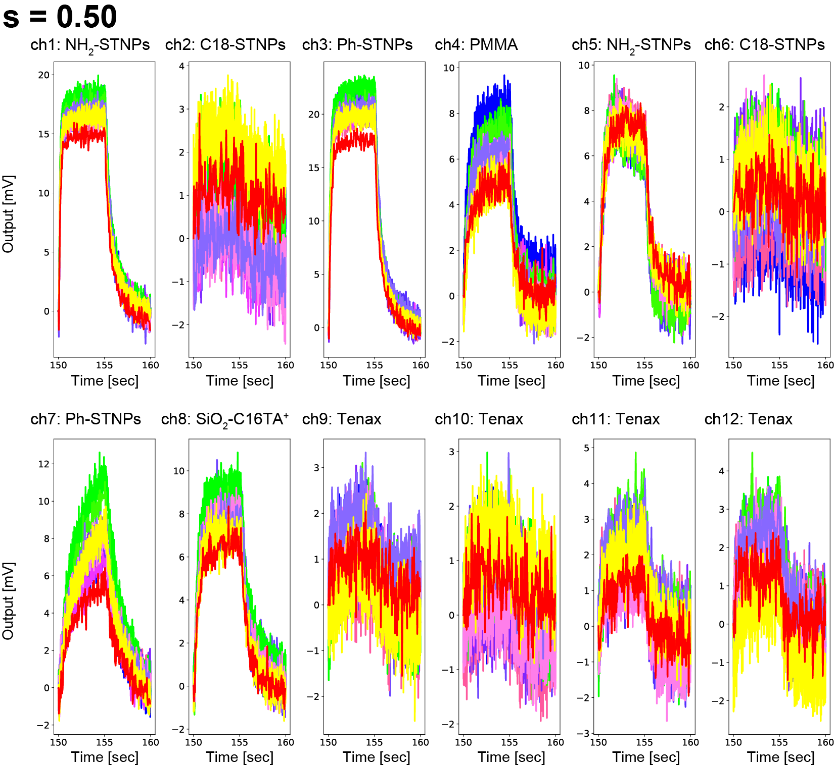
**

**Figure S2**. Response signals adding Gaussian noise with mean of 0 and standard deviation of $s=0.5$ obtained from 12 channels.

**
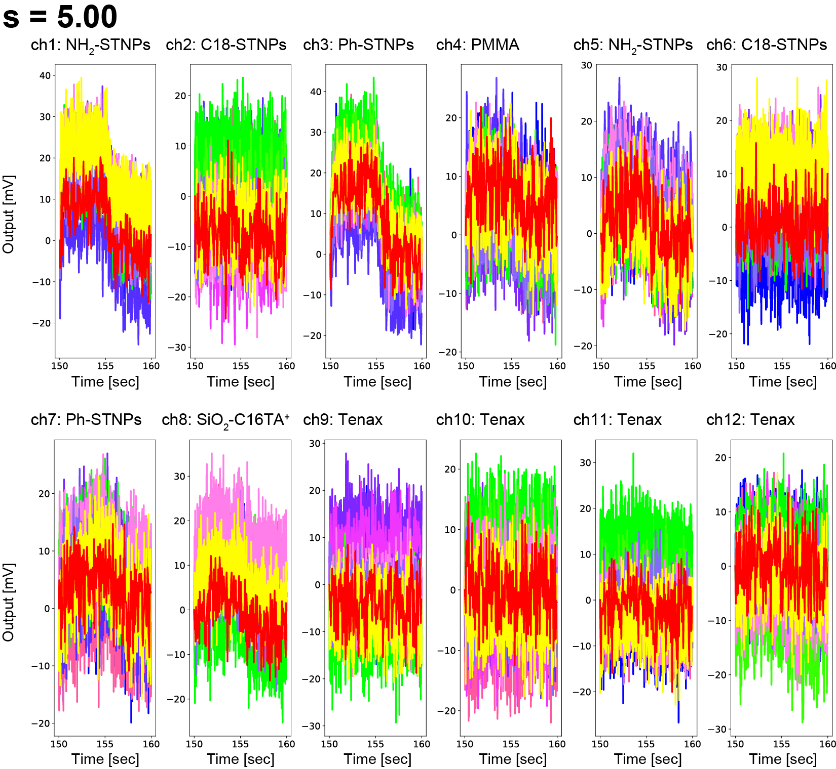
**

**Figure S3**. Response signals adding Gaussian noise with mean of 0 and standard deviation of $s=5.0$ obtained from 12 channels.


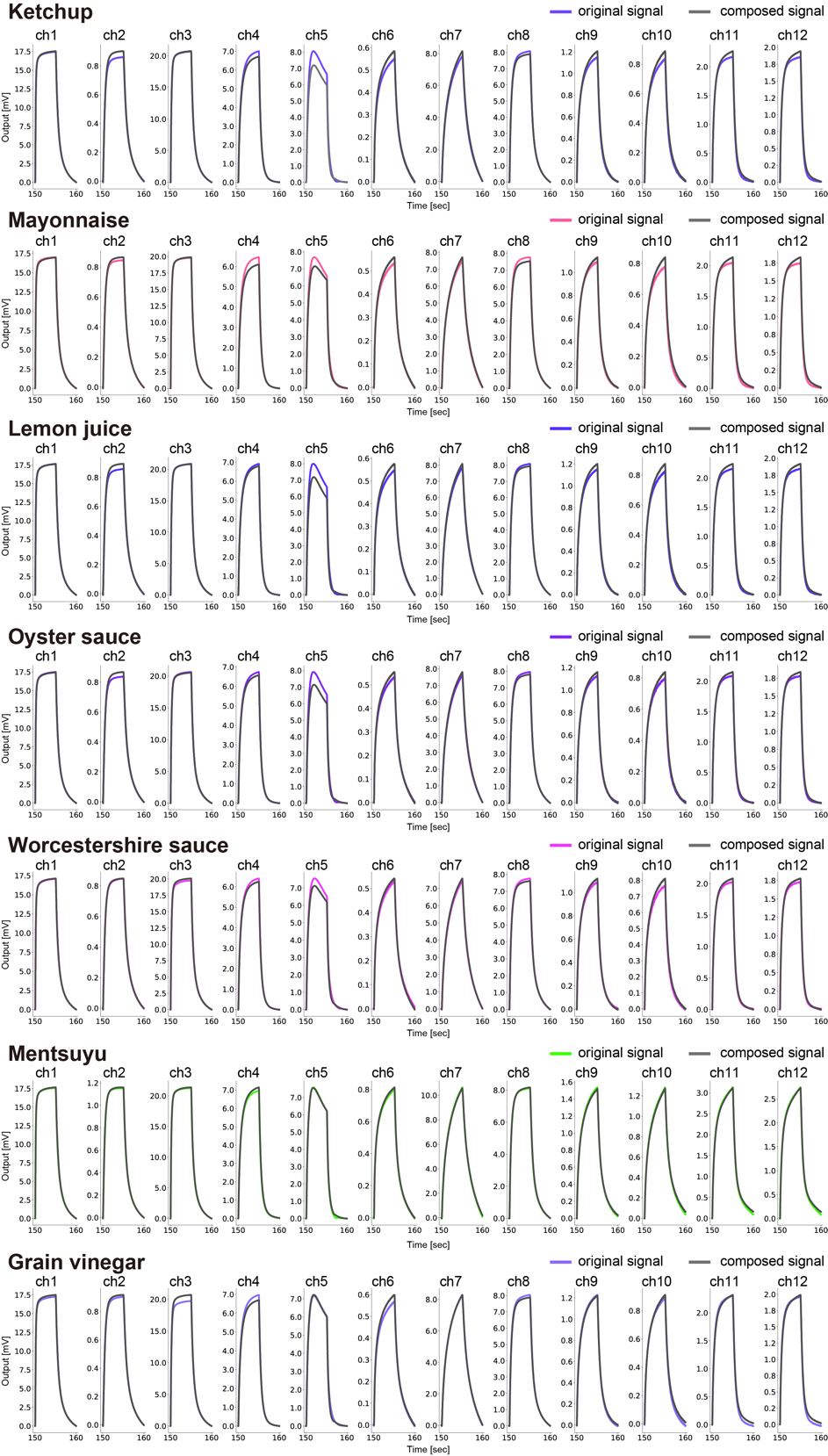


**Figure S4.** Comparisons between the original signals and the composed signals based on fish sauce, cooking sake, and water using the coefficients $(w_{1}, w_{2}, w_{3})$ for seven seasonings.


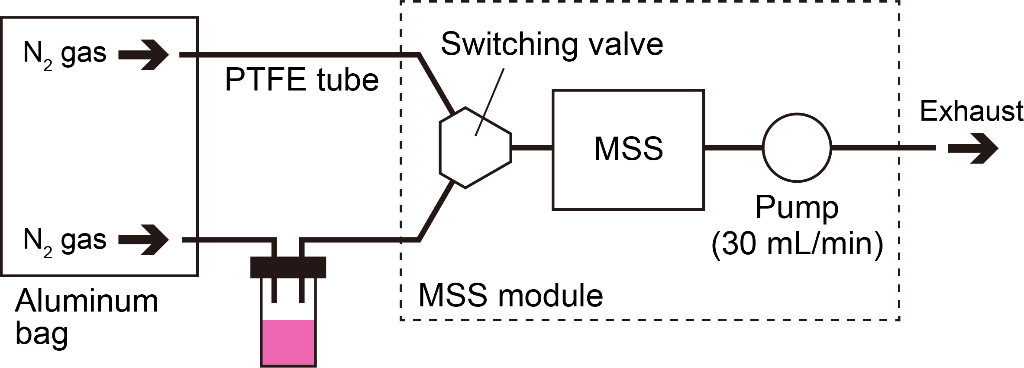


**Figure S5.** Schematic illustration of MSS measurement set-up.

**Table S1.** Ingredients in the present 11 seasonings.


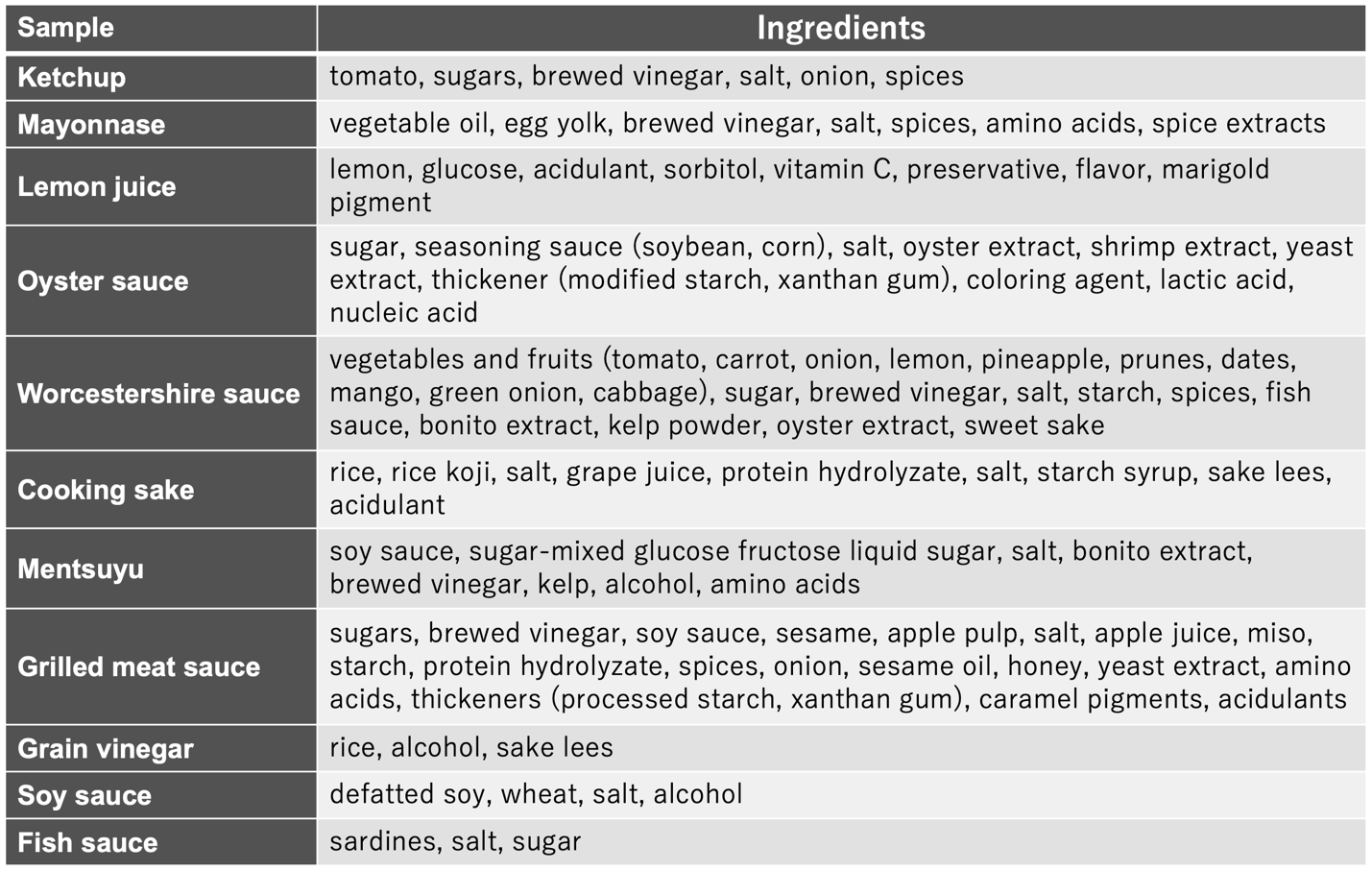


**Table S2.** EP score, concentration of primary odors $(w_{1}, w_{2}, w_{3}, w_{4})$, difference $\Delta$, and color for each odor sample when the four samples (fish sauce, cooking sake, pure water, and mentsuyu) are selected as quasi-primary odors.


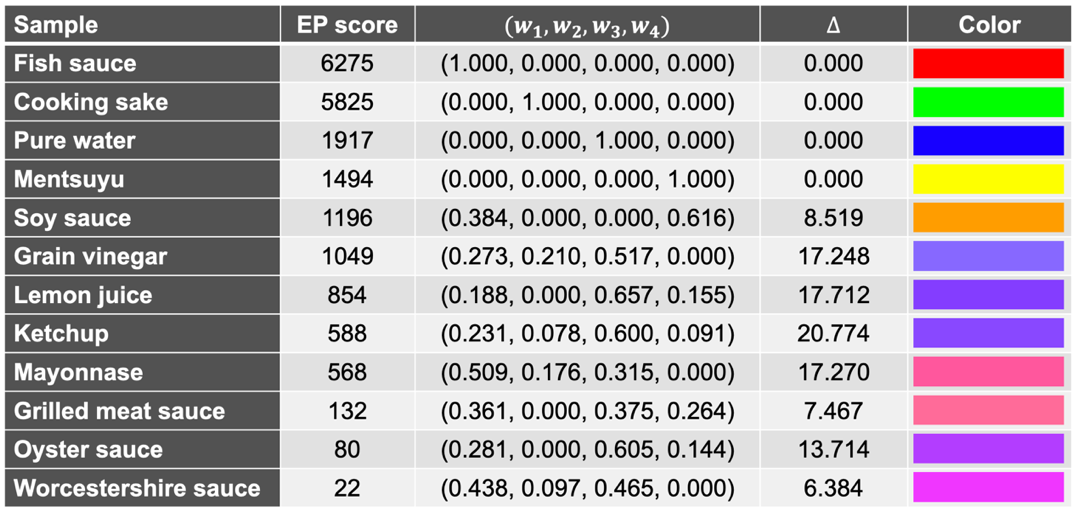


**Table S3.** Amounts of each chemical used for the synthesis of NH_2_-STNPs, C18-STNPs, and Ph-STNPs.


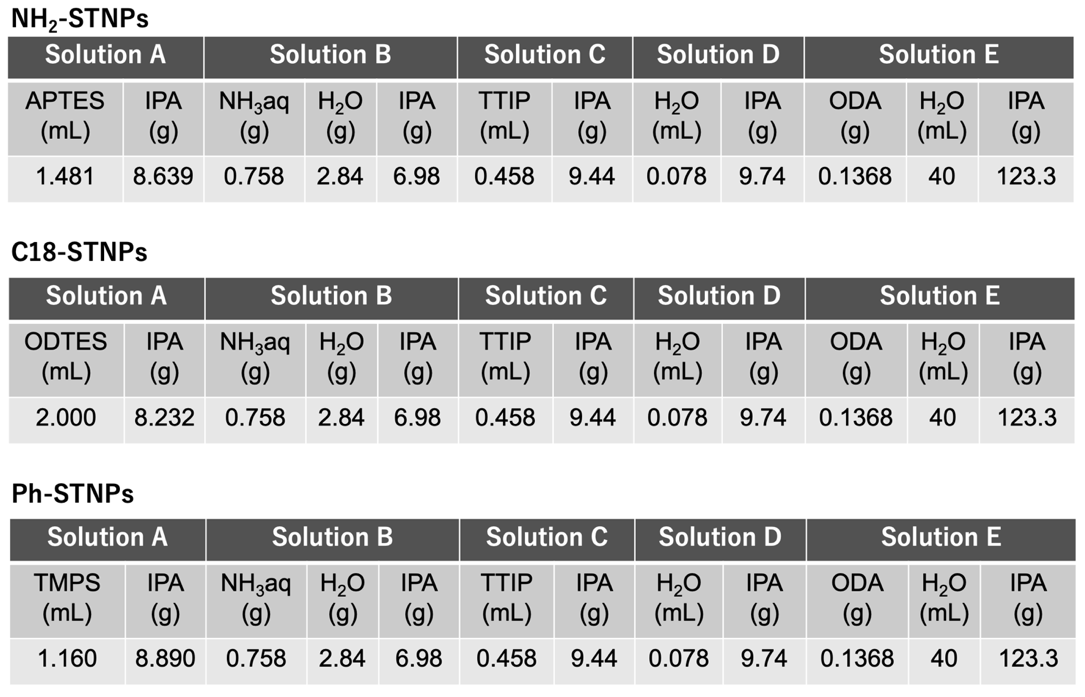


**Table S4.** Detailed coating conditions of receptor materials.


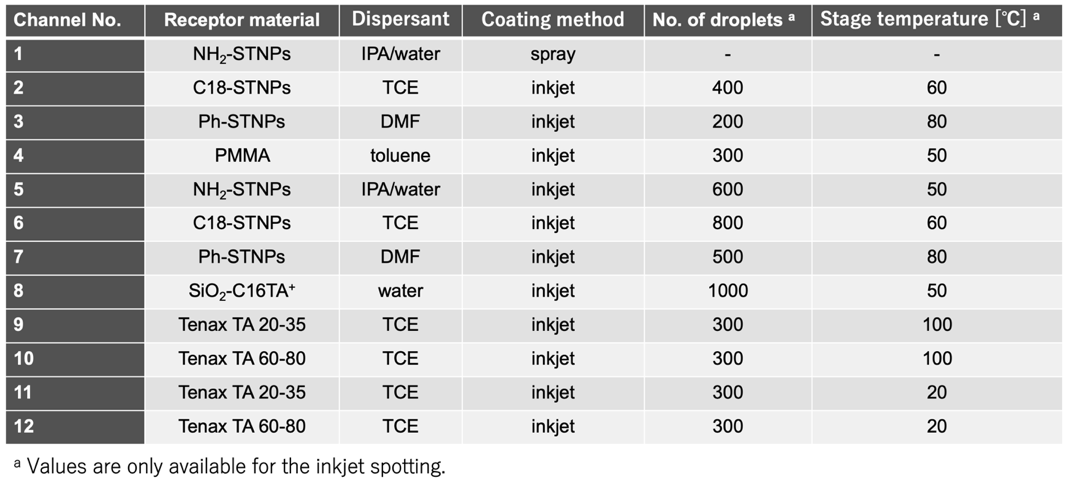


**Supporting video 1.** Outputs on our developed compact portable device when the real time color conversion is performed in the following order: fish sauce, cooking sake, pure water, grilled meat sauce, soy sauce, and three mixtures consisting of fish sauce and cooking sake with different concentrations (4:1, 2:1, and 1:1).
